# A Bicycle-Based Field Measurement System for the Study of Thermal Exposure in Cuyahoga County, Ohio, USA

**DOI:** 10.3390/ijerph13020159

**Published:** 2016-01-26

**Authors:** Nicholas B. Rajkovich, Larissa Larsen

**Affiliations:** 1 School of Architecture and Planning, University at Buffalo, 114 Diefendorf Hall, Buffalo, New York, NY 14214, USA; 2 Taubman College of Architecture and Urban Planning, University of Michigan, 2000 Bonisteel Boulevard, Ann Arbor, MI 48109, USA; larissal@umich.edu

**Keywords:** urban heat island, heat wave, heat health, thermal exposure

## Abstract

Collecting a fine scale of microclimate data can help to determine how physical characteristics (e.g., solar radiation, albedo, sky view factor, vegetation) contribute to human exposure to ground and air temperatures. These data also suggest how urban design strategies can reduce the negative impacts of the urban heat island effect. However, urban microclimate measurement poses substantial challenges. For example, data taken at local airports are not representative of the conditions at the neighborhood or district level because of variation in impervious surfaces, vegetation, and waste heat from vehicles and buildings. In addition, fixed weather stations cannot be deployed quickly to capture data from a heat wave. While remote sensing can provide data on land cover and ground surface temperatures, resolution and cost remain significant limitations. This paper describes the design and validation of a mobile measurement bicycle. This bicycle permits movement from space to space within a city to assess the physical and thermal properties of microclimates. The construction of the vehicle builds on investigations of the indoor thermal environment of buildings using thermal comfort carts.

## 1. Introduction

Over the last century, the average air temperature in the United States has increased by 0.7 °C to 1.1 °C [1]. While this increase should be of concern, and spur international action to reduce greenhouse gas emissions, air temperatures in a city are frequently 1.0 to 3.0 °C warmer than rural locations [2,3]. In addition, temperatures can vary by several degrees Celsius among the neighborhoods of a city because of variations in impervious surfaces, vegetation, and waste heat from vehicles and buildings [4,5]. While data taken by airport weather stations are reliable indicators of regional weather patterns, and are often used as the basis for heat health warning systems [6], these data are not necessarily representative of microclimatic conditions [7].

While techniques like remote sensing can provide block-level estimates of land surface temperature, this information is less useful for estimating human exposure to air temperature, a critical variable for planning public health responses to heat wave events [8]. Therefore, collecting a fine scale of microclimatic data is necessary to establish how physical characteristics (e.g., solar radiation, albedo, sky view factor, vegetation) contribute to local variations in exposure. Collecting these data suggest how urban design strategies (e.g., shading, light colored pavements, street tree planting) can reduce human exposure to temperature on hot days (>27 °C) [9].

The following paper describes the design and construction of the bicycle system, explains the testing and validation procedures used during the summer of 2012, and presents results from Cuyahoga County, Ohio, USA. Cleveland and its suburbs are the focus of this research because several national-level assessments of heat vulnerability identified the region as being extremely susceptible to high temperatures due to significant quantities of impervious surfaces, a lack of tree cover, a low percentage of homes with air-conditioning, a high rate of poverty, and an aging population [10,11,12].

The introduction is organized into three sections. The first part discusses the link between the physical characteristics of the built environment and increased ground surface and air temperatures. The second portion describes the use of mobile measurement systems to analyze thermal comfort and the urban canopy layer. The third and final section of the introduction introduces the research questions that guided the research.

### 1.1. Urban Heat Islands

Since the 1830s, studies in Europe, North America, and Asia have investigated the urban heat island (UHI) effect [13], though the first article to use the term “heat island” was not published until 1958 [2,14]. UHIs are broadly defined as the temperature difference between urbanized areas and their rural surroundings [15]. UHIs are a byproduct of all human settlements regardless of their size; they are an important topic for research because they increase temperature exposure during heat waves, increase electrical demand associated with air-conditioning, and increase ground-level smog [16].

This research focuses on ground surface and urban canopy layer UHIs because they increase human thermal exposure during extreme heat events. The following subsections outline how these UHIs form, measurement techniques, and their impacts on the urban climate [17].

#### 1.1.1. Surface Heat Islands

Ground surface heat islands are primarily a daytime phenomenon that form under several conditions: (1) when the albedo of land covers is reduced (e.g., when vegetated area is converted to concrete); (2) when thermal properties of materials increase storage of heat during the daytime; (3) when pollution creates a greenhouse effect that reduces radiative losses; and (4) when urban canyons decrease longwave radiation loss at night [16]. According to Oke [18], these ground surface heat islands are important to the overall energy balance of the urban climate because they modify air temperatures in the urban canopy layer, exchange energy with the lowest layers of the atmosphere, and directly impact human thermal by increasing mean radiant temperature.

Ground surface heat islands are typically measured by remote sensing equipment like satellites. For example, Lo and Quattrochi [19] used the Landsat visible, near infrared, and thermal wavelength data to develop statistical relationships between vegetation and ground surface temperatures in Atlanta. They found that surface temperatures and the normalized differential vegetation index (NDVI) were negatively correlated, which suggests that the concrete and asphalt that replaced forest and cropland had increased ground surface temperatures. Using Landsat, IKONOS, and Aqua satellite-based datasets, Imhoff *et al.* [2] found that impervious surface area explains 70% of the total variation in land surface temperature for thirty-eight of the most populous cities in the United States.

Under calm, cloudless conditions, the average difference in daytime surface temperatures between urban and rural sites is 10 to 15 °C; the difference in nighttime surface temperatures is less at 5 to 10 °C [15]. The magnitude of ground surface UHIs varies seasonally due to changes in the sun’s altitude, weather conditions, and vegetative cover. Surface UHIs are typically the greatest in the summer [18]. Although the primary impact of ground surface heat islands is a warming of the air, they also increase radiative gain to buildings, thereby increasing air-conditioner usage.

#### 1.1.2. Urban Canopy Layer Heat Islands

Urban canopy layer heat islands form under similar conditions to ground surface UHIs [20]. However, two additional factors cause localized warming of the air: (1) anthropogenic heat is released by the combustion of fuels from mobile and stationary sources; (2) a reduction of evaporating surfaces puts more energy into sensible rather than latent heat [16]. Canopy layer heat islands are the temperatures directly related to human temperature exposure occurring from roughly one meter above the ground to the average height of the surrounding buildings.

Measurement of air temperature in the urban canopy layer is difficult because most developed areas do not conform to standard guidelines for site selection and instrument exposure [7]. Of particular concern is the impact of local waste heat sources like building air-conditioning equipment, industrial sites, or vehicle exhaust if the goal is a representative measurement for a large area [4]. Recent efforts by Stewart (2011) have encouraged authors to report critical information about equipment type, calibration, and screen height to facilitate cross-comparison of studies; newly developed land cover classifications will also help to accurately define the source area of sensors. For stationary measurements of the canopy layer, most studies use either a weather station or microdataloggers to make observations of the local air temperature.

Canopy layer UHIs are weak during the day but become stronger after sunset due to the release of stored heat from the built environment. The timing and intensity depends on the season, prevailing weather conditions, and the properties of urban surfaces. Canopy layer heat islands are less intense than surface heat islands; air temperatures are on average only 1–3 °C warmer than in rural locations in temperate cities [2,3].

### 1.2. Mobile Measurement of the Thermal Environment

Data from airport weather stations do not have the resolution necessary to support microclimate studies at the district or neighborhood scale. While remote sensing can provide data on ground surface temperatures, vegetation levels, and albedo, limitations of aerial imagery include timing, cost, and spatial resolution. Additional fixed weather stations are expensive, are difficult to site, require permission for installation, are subject to vandalism and theft, and cannot be deployed quickly to capture data from a heat wave [7]. Due in part to these limitations, researchers have developed mobile measurement systems to analyze human thermal comfort and the urban canopy layer.

#### 1.2.1. Human Thermal Comfort

Moving measurement equipment from space to space on wheels, also called a “thermal comfort cart”, is a simple way to gather data from inside buildings to assess human thermal comfort. At one end of the cost spectrum, Kwok [20] describes the use of a simple push cart to transport handheld tools to evaluate thermal comfort in tropical classrooms. At the other end, Benton and colleagues [21] describe a portable thermal comfort field measurement system that gathered inputs from more than ten thermal environmental sensors and stored the results to a laptop.

While the complexity of these systems varies, the results from field studies of thermal comfort are comparable to one another because they typically reference American National Standards Institute /American Society of Heating, Refrigerating, and Air-Conditioning Engineers (ANSI/ASHRAE) Standard 55 to determine transducer height, accuracy, and response time [22]. However, a review of building thermal comfort research has revealed a lack of information on human response to conditions in semi-conditioned transitional spaces like passageways, courtyards, atria, and arcades. To address this gap, Potvin [23] investigated urban arcades using a backpack-based system, comparing thermal environmental conditions to established thermal comfort models. Building on this research, Chun and Tamura [24] used a cart to ferry equipment through a shopping mall, department store, and train station in Japan while surveying subjects. Their research showed that mobile methods could be extended to cover larger distances (~1 km) and semi-conditioned spaces, helping to bridge a gap in research between the indoor and outdoor thermal environments.

#### 1.2.2. Urban Canopy Layer

Mobile measurement of the urban canopy layer provides a simple way to gather data along a transect that spans urban and rural land uses. Mobile surveys are commonly used in urban climate studies to assess air temperature within canopy layer UHIs [25,26]; they can also be used as part of a larger observation network [27]. Automobiles, vans, or light trucks are the most common platform for these studies; temperature sensors are typically attached in front of the engine or to the roof or to avoid thermal contamination [28,29]. Advantages of mobile surveys include high temporal resolution of data, low cost compared to the expense of installing multiple stationary weather stations, and no need to cross-calibrate sensors and data from multiple sites [30].

Although using carts to study indoor thermal comfort and using automobile transects to analyze the urban canopy layer are both well-established approaches within their respective literatures, until recently few efforts bridged the gap between these micro- and mesoscale methods. Melhuish and Pedder [31] were the first to demonstrate that UHIs could be measured at very little cost by using handheld equipment transported by bicycle. Heusinkveld *et al.* [32] constructed a high-end mobile platform on a Dutch cargo tricycle to measure mean radiant and air temperatures in Rotterdam; their data were used to validate a thermal comfort model [33].

Brandsma and Wolters [34] split the difference between these two approaches by collecting air temperature data using a microdatalogger attached to the front of a bicycle. Over a three-year period, they collected data along a 14-kilometer-long transect in Utrect to describe the magnitude of the local UHI. They then used a regression model to predict the mean and maximum UHI intensity using local land covers as independent variables. This study had the greatest influence on this research; it showed how a bicycle could be used to obtain high-resolution observations of the UHI along a transect.

Finally, Coseo [9] used an industrial tricycle to transport a full weather station with a telescoping three-meter measurement mast to measure the microclimates of eight neighborhoods in Chicago, Illinois. While all of these studies demonstrate the utility of using cycles to take measurements, the methodology is still evolving, and their remains challenges such as accounting for the movement of the equipment during testing. The following sections of this paper present the use of a bicycle to gather ground and air temperature data along an urban-to-rural transect in Northeastern Ohio.

## 2. Experimental Section

Cuyahoga County is located in the humid, mid-latitude zone of the United States at approximately 41.5°N and 81.7°W. The average temperature in July is 23.1 °C with a high of 28.1 °C; the average temperature in January is −2.2 °C with a low of −5.7 °C [35].

The northern border of Cuyahoga County is Lake Erie; this large body of freshwater moderates temperatures in the height of summer. Although this heat sink provides a protective effect for Greater Cleveland, the frequency of heat waves in the Midwest has increased over the last sixty years [36]. In addition, the magnitude of heat stress in the region is projected to grow because of local increases in humidity [37]. Therefore, understanding how factors like land cover moderate local temperatures is an important first step in reducing heat-related morbidity and mortality.

### 2.1. Mobile Measurement System Design

Although other studies have used bicycles as a platform to analyze the urban canopy layer, these studies gathered air temperature and relative humidity with either handheld equipment or microdataloggers attached to the bicycle. To the best of our knowledge. this is the first time a research-grade weather station has been installed on a bicycle to gather multiple types of data (e.g., ground surface temperature, solar radiation, sky view factor) for analysis. A secondary goal was determining the amount of equipment that could be carried, whether a bicycle was a suitable platform for this type of analysis, and what limitations non-motorized transportation imposed on the investigation of rural-to-urban transects.

Because meteorological equipment needed to be at least 1.25-meters above grade to avoid interference from the ground [7], and the equipment needed to be moved up to 50 kilometers per transect, a cargo bicycle was chosen as a base for the equipment. Cargo (or trekking) bicycles are commonly used for bicycle touring with large amounts of camping equipment. They differ from standard bicycles in that they have a heavier-duty frames, spokes, and brakes, as well as longer wheelbases to improve stability under load.

**Figure 1:**
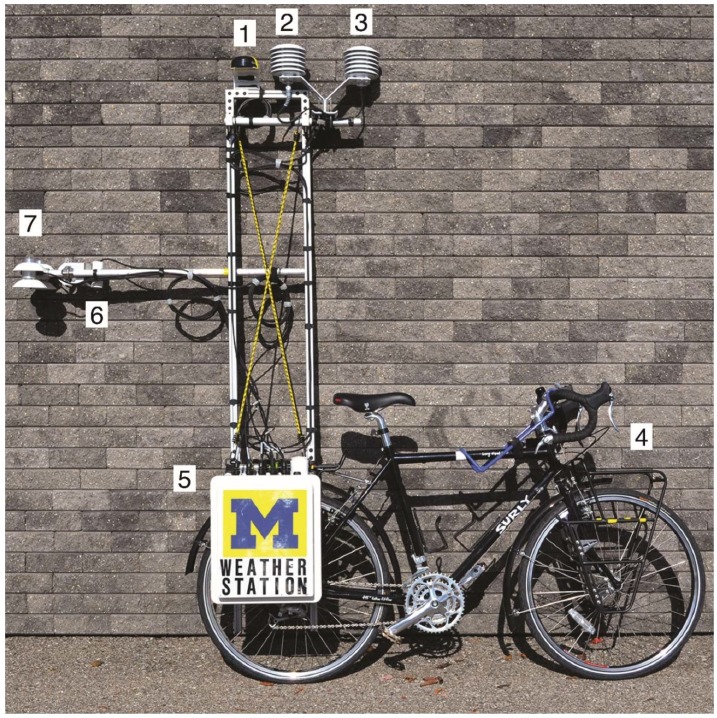
Image of the bicycle-based mobile measurement system: (**1**) GPS; (**2**) relative humidity and air temperature; (**3**) air temperature; (**4**) sky view factor camera location (not shown); (**5**) datalogger and barometric pressure; (**6**) ground surface temperature; (**7**) four component net radiometer.

Figure 1 illustrates the final configuration of the equipment on the cargo bicycle; Table 1 presents the specifications for each piece of equipment. A thermocouple unit, hygrometer unit, and GPS unit were installed at the top of a 2.0-meter-tall aluminum tower constructed of extruded aluminum sections. The GPS unit collected latitude, longitude, and speed, and provided a time stamp to synchronize fisheye images taken by a camera to determine sky view factor. A four component net radiometer and infrared radiometer were installed off the back of the bicycle 1.25 meters above the ground to gather information about incoming and outgoing short- and longwave radiation and ground surface temperature. All of the equipment took a reading once every second; the datalogger averaged the measurements for each minute and stored it to an onboard solid state hard drive.

The aluminum bar installed horizontally off of the back of the bicycle was an attempt to move the net radiometer and infrared radiometer as far away as possible from the bicycle to reduce interference; a 1.0-meter distance balanced the bicycle weight from front to back to prevent tipping when ascending or descending hills. A bicycle trailer was not suitable for this purpose because the equipment might be damaged on rough pavement (the net radiometer contained a platinum fine wire thermistor that could easily be damaged). A watertight box contained the datalogger and a barometer to measure atmospheric pressure. Both the tower and datalogger box were connected to a bicycle rack using standard pannier hardware that facilitated quick assembly on site.

**Table 1:** Equipment installed on the mobile measurement system.

	Description	Location	Accuracy
Air Temperature	BetaTherm 100K6A1IA Thermistor in 6-Plate Radiation Shield	Top of mast, 2 m	±0.2 °C
Relative Humidity	Campbell CS215	Top of mast, 2 m	±0.2 °C, ±4% RH
Incoming/Outgoing Solar Radiation	Hukseflux NR01 Net Radiometer	Off back of mast, 1.25 m	±2.5%, ISO Second Class
Incoming/Outgoing Longwave Radiation			
Ground Surface Temperature	Apogee SI-111 Infrared Radiometer	Off back of mast, 1.25 m	±0.2 °C
Latitude/Longitude	Garmin GPS16X-HVS	Top of mast, 2 m	<3 m with DGPS correction
Time			1 µs
Wind Speed	N/A		
Barometric Pressure	Vaisala PTB110	In datalogger enclosure	±0.3 hPa at +20 °C
Sky View Factor	Nikon D5100 w/Sunex 5.6 mm Fisheye Lens	Front Bicycle Rack	Not Specified
	Nikon GP-1		
Datalogger	Campbell CR3000	Rear Bicycle Rack	Varies by Input Type
Datalogger Enclosure	Campbell PWENC 12/14		N/A
**Total Weight**	**Bicycle: ~15 kg, Equipment: ~30 kg**		

Table format adapted from Benton *et al.* [21]. Data provided by Campbell Scientific, Inc., and individual sensor manufacturers.

#### Sky View Factor Measurement

A common parameter used to characterize the geometry of urban canyons is the sky-view factor (SVF), a measure of the degree to which the sky is obscured by the surroundings for a given point. Several authors have described the use of fisheye lenses for measurement of SVF [38,39], and Frazer *et al.* [40] described the advantages of digital- over film-based systems. However, the design of this SVF measurement system was most influenced by Grimmond *et al.* [41], who used a digital camera with a fisheye lens to gather SVF data in Bloomington, Indiana.

This system used a newer version of the same Nikon camera, the D5100, which had an on-board intervalometer to take an image every minute. In addition, an accessory GPS unit synchronized the photography with the datalogger data collection cycle and geocoded each image. A fixed f/5.6 Sunex 5.6 mm fisheye lens was used for imagery because it had a 185° field of view and no moving parts that could be damaged by vibration.

The photographic equipment was placed in an air- and watertight plastic box with a clear acrylic dome on the top. The plastic case contained protective foam in case the apparatus fell off the bike. In the box on the front of the bike, the camera lens was 0.75 meters above the ground. As the lens had a high fixed f-stop, every image was in focus and had a good depth of field with a shutter speed of 1/1000 s.

Because the camera was facing toward the sky, solar radiation tended to overheat the interior of the box and the camera. A reusable icepack was chilled in a refrigerator and placed in the box prior to each transect to keep the camera cool. In addition, white electrical tape was placed around the base of the clear plastic dome to reflect as much solar radiation as possible without obstructing the image.

The camera collected a hemispherical image every minute of the transect. After each ride, it was necessary to crop out the half of the image that was obscured by the rider and equipment. A computer code was developed to automate the cropping, reduce the file size, and convert the image to black and white for analysis. After all of the images were processed, they were input into the SkyViewFactor Calculator software, version 1.1 [42]. Using the time and geocode on each image, it was possible to match the SVF data with the other meteorological data gathered by the bicycle.

### 2.2. Route Selection

**Figure 2:**
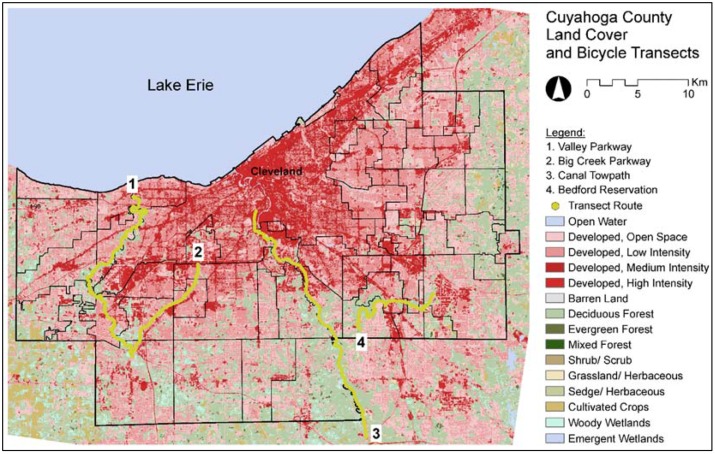
Map of transect routes. Data sources: Cuyahoga County Geographical Information Systems Department (2012), U.S. Census Bureau (2010), National Land Cover Database (2006).

To test the limitations of the bicycle, a number of bicycle paths in Cuyahoga County were selected with a variety of land covers, topographies, distances from Lake Erie, and paving types. The Cleveland Metroparks and the Cuyahoga Valley National Park maintained the bicycle paths; both organizations required research permits and liability insurance prior to the first ride. Based on a walk-through of each site, the transects were limited to the four paths presented in Figure 2.

Because one goal of the research was to correlate air temperature with land cover (*i.e.*, impervious surfaces, bare soil, grass, forest, and water) the bicycle was used on days immediately before or after a Landsat 7 acquisition date. However, the data was post-processed, cloud cover and gaps in the data made the satellite information unusable. A 10-meter resolution land cover analysis recently completed by the Cuyahoga County Geographical Information Systems (GIS) Department [43] was substituted for the satellite data.

To randomize the collection of data, a random number table was used to select the path and direction for each ride. Every attempt was made to ride the paths during the hottest part of the day, typically in the late afternoon. Due to safety concerns, the bicycle was not operated at night. In total, twelve rides were completed on the bicycle paths where the data was deemed usable.

### 2.3. Data Quality Control and Reference Air Temperatures

For the air temperature analysis, it was necessary to have an air temperature from a local station to control for daily weather conditions. Data was used from site CND01 because it recorded measurements every 6 min, limiting the need for interpolation between data points.

According to the NOAA National Data Buoy Center website, the CND01 weather station is located at 41.542°N and 81.637°W. Air temperature on site is measured at 3.9 m above the ground; wind speed is gathered at 7.77 m above the ground [44]. Before using data from this weather station, a site visit was conducted to ensure that the measurement equipment was not subject to contamination from local heat sources like automobile exhaust or air-conditioning equipment.

### 2.4. Data Processing

The data from the bicycle was linked with the reference air temperature data from station CND01 using the timestamp. This data was input into GIS software to begin examining spatial relationships among the data. Using the latitude and longitude data associated with each measurement point, the data points were geolocated on the Canal Towpath and then overlaid onto the 10-meter resolution land cover from the Cuyahoga County GIS Department.

Local ground and air temperatures under the canopy layer are affected by land covers; this is frequently called the “source area” or “footprint” of the measurement [7]. Source areas are often assumed to have a circular or elliptical shape [4,34]. To facilitate comparsion of the results with those of Brandsma and Wolters [35] and Coseo and Larsen [4], circles were selected with radii varying from 25 to 600 m. An ellipse was also selected that was 500 m long and 300 m wide, with the long axis of the ellipse facing upwind at each point. GIS software was then used to extract the fraction of each of the five land covers around each measurement point.

## 3. Results and Discussion

Because of the day-to-day variability of weather, it was necessary to control for local conditions and unobserved phenomena during each ride. To this end, several control variables were included in the analysis. These variables included the reference air temperature from station CND01, the speed of the bicycle, dummy variables for each of the four rides, and five latitude and longitude variables to account for spatial autocorrelation.

Before performing a statistical analysis, a bivariate analysis was conducted to understand the relationship among the independent variables and the ground surface temperature on the four rides on the towpath. Table 2 presents the correlation among these variables. The 11 significant bivariate correlations (*p* < 0.001) were (1) reference air temperature (0.130); (2) incoming solar radiation (0.696); (3) albedo (−0.308); (4) sky view factor (0.297); (5) dummy variable for the second ride on 25 July 2012 (0.253); (6) dummy variable for the third ride on 25 July 2012 (−0.297); (7) latitude (0.260); (8) longitude (−0.234); (9) latitude to the second power (0.260); (10) longitude to the second power (0.234); and (11) latitude multiplied by longitude (−0.255). Neither the dummy variable for the fourth ride nor the speed of the bicycle were statistically significant. The bivariate correlations help to clarify the relationship among variables before proceeding to a regression model.

Although there was high correlation among all five of the latitude and longitude variables, they are only used as a control for spatial autocorrelation and are not statistically significant in the final regression model. Any inflation in the variance and standard error would be limited to these spatial variables. While several of these variables could have been omitted to reduce multicollinearity among the spatial variables—for example, by using only latitude and longitude as a control—it was important to include latitude and longitude to the second power because the variation in observations might not vary linearly. Latitude multiplied by longitude was included to account for any interactive effect between latitude and longitude, since the towpath route is oriented NNW to SSE. This approach is consistent with those of other studies that include spatial variables in regression models, for example Immergluck and Smith [45] and Galster *et al.* [46].

**Table 2:** Bivariate correlations among physical characteristics and ground surface temperature.

	Ground Surface Temp.	Reference Air Temp.	Solar Rad.	Albedo	SVF	Bicycle Speed	Ride 2 Dummy	Ride 3 Dummy	Ride 4 Dummy	Lat.	Long.	Latitude^2^	Longitude^2^
Reference Air Temp.	0.130 ***												
Solar Rad.	0.696 ***	−0.164 ***											
Albedo	−0.308 ***	0.529 ***	−0.123 ***										
SVF	0.297 ***	−0.309 ***	0.417 ***	−0.292 ***									
Bicycle Speed	−0.005	−0.201 ***	0.045	−0.229 ***	0.161 ***								
Ride 2 Dummy	0.253 ***	0.416 ***	0.318 ***	0.437 ***	−0.208 ***	−0.252 ***							
Ride 3 Dummy	−0.297 ***	0.108 **	−0.095 **	−0.024	−0.012	0.102 **	−0.400 ***						
Ride 4 Dummy	−0.060	−0.857 ***	0.135 ***	−0.541 ***	0.341 ***	0.199 ***	−0.340 ***	−0.309 ***					
Latitude	0.260 ***	−0.619 ***	0.209 ***	−0.614 ***	0.491 ***	0.225 ***	−0.375 ***	−0.237 ***	0.687 ***				
Longitude	−0.234 ***	0.655 ***	−0.186 ***	0.625 ***	−0.444 ***	−0.196 ***	0.380 ***	0.263 ***	−0.733 ***	−0.977 ***			
Latitude^2^	0.260 ***	−0.619 ***	0.209 ***	−0.614 ***	0.491 ***	0.224 ***	−0.375 ***	−0.237 ***	0.687 ***	1.000 ***	−0.977 ***		
Longitude^2^	0.234 ***	−0.655 ***	0.186 ***	−0.625 ***	0.444 ***	0.196 ***	−0.380 ***	−0.263 ***	0.733 ***	0.977 ***	−1.000 ***	0.977 ***	
Lat. × Long.	−0.255 ***	0.632 ***	−0.204 ***	0.619 ***	−0.481 ***	−0.218 ***	0.378 ***	0.245 ***	−0.703 ***	−0.998 ***	0.987 ***	−0.998 ***	−0.987 ***

** *p* < 0.01, *** *p* < 0.001.

### 3.1. Ground Surface Temperature Regression Analysis

Drawing on the results of the bivariate correlation and a review of the literature, the hypothesis was that incoming solar radiation, albedo, and sky view factor would explain the variation in ground surface temperatures along each transect. To test this hypothesis, an ordinary least squares (OLS) regression was performed to determine the explanatory power of the independent variables on ground surface temperature.

The hypothesis was tested with three models. Model 1 included only the independent variables and a control for bicycle speed. Model 2 utilized all of the variables from the first model and added three dummy variables to control for unobserved phenomena on each ride. Model 3 included five spatial variables based on the latitude and longitude of each observation to control for spatial autocorrelation. Results from the regression analysis appear in Table 3.

Model 1, which includes reference air temperature, incoming solar radiation, albedo, sky view factor, and the bicycle speed, explains 71.8% of the variation in ground surface temperature. The model is statistically significant, with an F-statistic of 382.64. (The F-statistic is a test statistic for linear regression models that helps with evaluating the statistical significance of a model and its components.) With the exception of sky view factor, all of the independent variables are statistically significant (*p* < 0.01).

Including the dummy variables for each of the four rides gives Model 2 an adjusted R^2^ of 0.845, an improvement of 0.127 over Model 1. This model is also statistically significant, with an F-statistic of 511.90. In the second model, all of the variables are significant except sky view factor and the control variable for bicycle speed (*p* < 0.001).

**Table 3:** Regression analysis for ground surface temperatures on the canal towpath.

	Model 1			Model 2			Model 3		
	β	SE	*t*−Statistic	β	SE	*t*−Statistic	β	SE	*t*−Statistic
Reference Air Temperature	1.300 ***	0.060	21.52	1.007 ***	0.078	12.91	1.023 ***	0.076	13.50
Incoming Solar Radiation	0.014 ***	0.000	33.32	0.016 ***	0.000	40.31	0.015 ***	0.000	40.57
Albedo	−36.350 ***	1.723	−21.09	−37.589 ***	1.367	−27.50	−34.011 ***	1.441	−23.61
Sky View Factor	0.367	0.415	0.89	0.180	0.326	0.55	−0.277	0.336	−0.83
Bicycle Speed	−0.168 **	0.065	−2.59	−0.082	0.049	−1.67	−0.068	0.049	−1.41
Ride 2 Dummy				−2.475 ***	0.239	−10.35	−2.011 ***	0.241	−8.34
Ride 3 Dummy				−5.162 ***	0.208	−24.73	−4.632 ***	0.217	−21.31
Ride 4 Dummy				−3.927 ***	0.389	−10.09	−4.839 ***	0.411	−11.78
Latitude							−2780.866	28945.53	−0.10
Longitude							3650.522	41717.6	0.09
Latitude^2^							143.972	247.177	0.58
Longitude^2^							50.716	380.544	0.13
Latitude × Longitude							111.713	581.365	0.19
Constant	7.363 ***	1.467	5.02	15.643 ***	1.861	8.41	205958.3	1222943	0.17
*n*	759			759			759		
F	382.64			511.90			340.32		
Adjusted R^2^	0.718			0.845			0.856		
Change in R^2^				0.127			0.011		

** *p* < 0.01, *** *p* < 0.001.

Adding the five latitude/longitude variables to control for spatial autocorrelation in Model 3 improves the adjusted R^2^ to 0.856, an improvement of 0.011 over Model 2. However, the F-statistic of the model drops to 340.32, likely because five additional variables that are not statistically significant were added to the model. In this third model, only the reference air temperature, solar radiation and albedo are statistically significant (*p* < 0.001), and they have similar coefficient values to Model 2. This may mean that spatial variables could be omitted from a ground temperature model; however, further analysis of additional rides would be necessary to test this claim.

**Figure 3:**
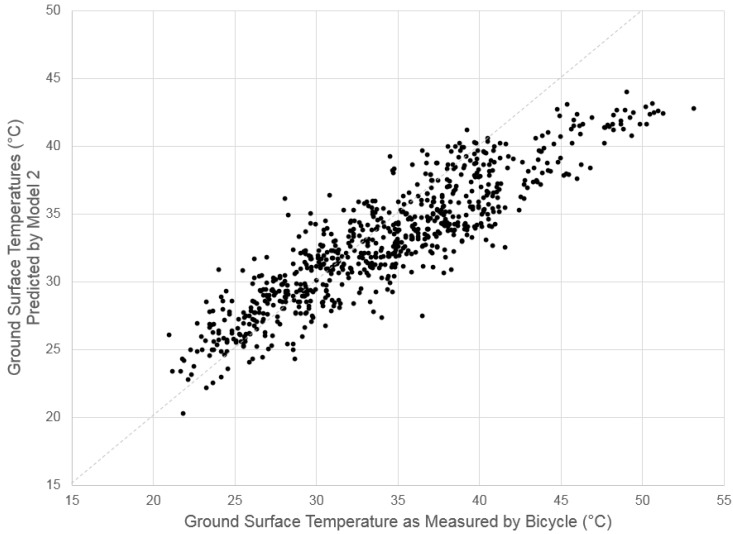
Comparison of ground surface temperature as measured by the bicycle and as predicted by Regression Model #2.

Figure 3 compares the ground surface temperature as measured by the equipment on the bicycle and as predicted by Model 2. From the lowest temperatures recorded (~20 °C) through approximately 40 °C, there is a strong fit in the model. Above 40 °C, Model 2 has a tendency to underpredict the ground surface temperature; this may mean that other factors such as soil type, soil moisture, pavement thickness, time of day, or other factors lead to higher temperature pavement.

### 3.2. Air Temperature Bivariate Correlations

Before performing a statistical analysis, a bivariate analysis was conducted to understand the relationship among the independent variables and the air temperatures on three rides on the towpath. The fourth ride was omitted from 16 September 2012 because the air temperatures were low; the primary interest was in the relationship among physical characteristics and air temperatures on hot days (>27 °C). Table 4 presents the correlations among these variables.

The fractions of forest and water were calculated from the Cuyahoga land cover database as the percentage of each land cover in an ellipse that was 500 m long and 300 m wide with the long axis oriented upwind from the observation [4,7]. While the Cuyahoga County land cover database characterized land covers for five types (impervious, barren, grass, forest, and water), impervious and grass land covers had a similar coefficient that increased air temperature. Very few of the observations (<5%) had bare soil in the upwind ellipse. Therefore, these three land covers (impervious, barren, grass) were folded into one classification called “impervious” for the analysis.

The 11 significant bivariate correlations (*p* < 0.025) were (1) the reference air temperature at station CND01 (0.734); (2) ground surface temperature (0.232); (3) fraction water (−0.142); (4) bicycle speed (−0.114); (5) dummy variable for the first ride on 27 June 2012 (0.199); (6) dummy variable for the second ride on 25 July 2012 (0.235); (7) latitude (−0.003); (8) longitude (−0.011); (9) latitude to the second power (−0.003); (10) longitude to the second power (−0.011); and (11) latitude multiplied by longitude (0.005). The fraction of forest in the upwind ellipse was not statistically significant. The bivariate correlations helped to clarify the relationship among variables before proceeding to a regression model.

**Table 4:** Bivariate correlations among physical characteristics and measured air temperature.

	Measured Air Temp.	Reference Air Temp.	Ground Surface Temp.	Fraction Forest	Fraction Water	Bicycle Speed	Ride 1 Dummy	Ride 2 Dummy	Lat.	Long.	Latitude^2^	Longitude^2^	Lat. x Long.
Measured Air Temp.	1												
Reference Air Temp.	0.734 ***	1											
Ground Surface Temp.	0.232 ***	0.050	1										
Fraction Forest	−0.090	0.111 *	−0.209 ***	1									
Fraction Water	−0.142 **	−0.026	−0.140 **	−0.119 *	1								
Bicycle Speed	−0.114 *	−0.117 *	−0.017	−0.181 ***	−0.005	1							
Ride 1 Dummy	0.199 ***	0.013	0.028	−0.189 ***	−0.058	0.010	1						
Ride 2 Dummy	0.235 ***	0.360 ***	0.316 ***	0.207 ***	−0.124 **	−0.220 ***	−0.507 ***	1					
Latitude	−0.003	−0.169 ***	0.470 ***	−0.402 ***	−0.305 ***	0.170 ***	0.160 ***	−0.041	1				
Longitude	0.011	0.157 ***	−0.487 ***	0.397 ***	0.268 ***	−0.160 ***	−0.209 ***	0.082	−0.976 ***	1			
Latitude^2^	−0.003	−0.169 ***	0.470 ***	−0.402 ***	−0.305 ***	0.169 ***	0.161 ***	−0.041	1.000 ***	−0.976 ***	1		
Longitude^2^	−0.011	−0.157 ***	0.487 ***	−0.397 ***	−0.268 ***	0.160 ***	0.209 ***	−0.082	0.976 ***	−1.000 ***	0.976 ***	1	
Latitude × Longitude	0.005	0.166 ***	−0.476 ***	0.402 ***	0.298 ***	−0.168 ***	−0.173 ***	0.051	−0.999 ***	0.986 ***	−0.999 ***	−0.986 ***	1

* *p* < 0.025, ** *p* < 0.01, *** *p* < 0.001.

### 3.3. Air Temperature Regression Analysis

Drawing on the results of the bivariate correlation and a review of the literature, reference air temperature, ground surface temperature, and land cover types were hypothesized to explain the variation in air temperatures along each transect. To test this hypothesis, an ordinary least squares (OLS) regression was performed to determine the explanatory power of the independent variables on air temperature. Because of the day-to-day variability of weather, it was necessary to control for local conditions and unobserved phenomena during each ride. To this end, several control variables were included in the analysis. These variables included reference air temperature from station CND01, the speed of the bicycle, dummy variables for each of the four rides, and five latitude and longitude variables to account for spatial autocorrelation.

The hypothesis was tested with three models. Model 1 included only the independent variables and a control for bicycle speed. Model 2 used all of the variables from the first model and added two dummy variables to control for unobserved phenomena on each ride. Model 3 included five spatial variables based on the latitude and longitude of each observation to control for spatial autocorrelation. The analysis was limited to temperatures over 27 °C because of an interest in the relationship among the independent variables and air temperature on the hottest days to help quantify exposure during heat waves. Results from the regression analysis appear in Table 5.

**Table 5:** Regression analysis for measured air temperature on the canal towpath.

	Model 1			Model 2			Model 3		
	β	SE	*t*−Statistic	β	SE	*t*−Statistic	β	SE	*t*−Statistic
Reference Air Temperature	0.490 ***	0.020	24.520	0.483 ***	0.021	22.590	0.479 ***	0.020	23.520
Ground Surface Temp.	0.014 ***	0.003	4.620	0.014 ***	0.003	4.330	0.020 ***	0.003	5.940
Fraction Forest	−0.476 ***	0.090	−5.320	−0.387 ***	0.090	−4.310	−0.249 *	0.104	−2.410
Fraction Water	−0.875 ***	0.215	−4.060	−0.771 ***	0.212	−3.640	−0.677 **	0.215	−3.150
Bicycle Speed	−0.022	0.012	−1.860	−0.019	0.012	−1.670	−0.013	0.011	−1.150
Ride 1 Dummy				0.171 ***	0.039	4.440	0.226 ***	0.037	6.070
Ride 2 Dummy				0.016	0.047	0.330	−0.009	0.045	−0.210
Latitude							11795.720	6127.608	1.930
Longitude							−25575.51 **	9797.343	−2.610
Latitude^2^							277.327 ***	57.064	4.860
Longitude^2^							−48.836	83.202	−0.590
Latitude × Longitude							425.646 ***	124.343	3.420
Constant	16.930 ***	0.483	35.050	16.931 ***	0.512	33.070	−1287504 ***	309841.4	−4.160
*n*	448			448			448		
F	139.73			109.51			80.31		
Adjusted R^2^	0.608			0.630			0.680		
Change in R^2^				0.022			0.05		

* *p* < 0.025, ** *p* < 0.01, *** *p* < 0.001.

Model 1, which includes reference air temperature, ground surface temperature, fraction forest, and fraction water, explains 60.8% of the variation in air temperature. With the exception of bicycle speed, all of the independent variables are statistically significant (*p* < 0.001). Including the dummy variables for each of the four rides improves the adjusted R^2^ of Model 2 to 0.630, an improvement of 0.022 over Model 1. However, the F-statistic declines from 139.73 to 109.51. In the second model, all of the independent variables are statistically significant; the control variable for bicycle speed and the dummy variable for the second ride are the only factors that do not achieve statistical significance at the 0.001 level.

Adding the five spatial variables to control for autocorrelation in Model 3 improves the adjusted R^2^ to 0.680, an improvement of 0.05 over Model 2. However, the F-statistic of the model drops further to 80.31, likely because five variables with limited statistical significance were added to the model. However, it was important to control for spatial autocorrelation because observations near one another could skew the direction of the coefficients and their value. In this third model, all of the independent variables are statistically significant at the 0.025 level.

**Figure 4:**
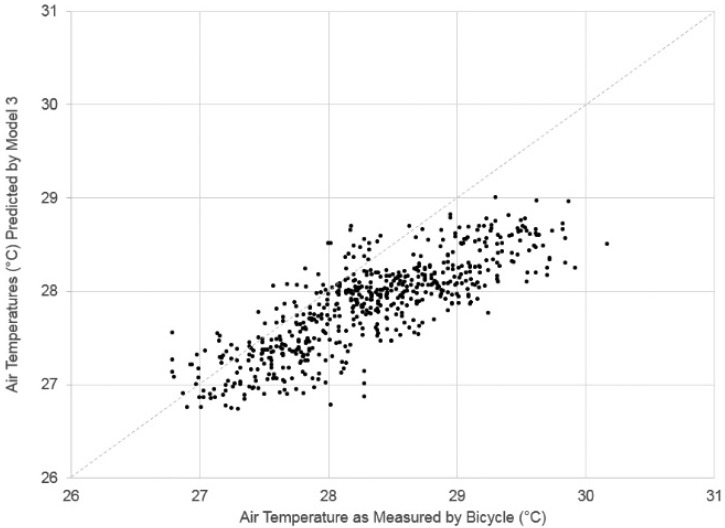
Comparison of air temperature as measured by the bicycle and as predicted by Regression Model #2.

Figure 4 compares the air temperature as measured by the equipment on the bicycle and as predicted by Model 3. Similar to the ground temperature regression models, this model has a tendency to underpredict the highest air temperatures. This is likely because air temperature can fluctuate in very short distances due to things like waste heat emissions while components of the regression model (*i.e.*, reference air temperature, land cover) change slowly over a transect.

#### Testing Diameters of Source Areas

Although the model presented in the last section used an upwind 500 m-by-300 m ellipse to quantify land cover, several radii of circles were tested around each observation point to determine their contribution to air temperature. This analysis was conducted to compare results with the findings of Brandsma and Wolters [35], the only other study that used a bicycle to gather air temperature data to quantify a canopy layer UHI.

Table 6 presents the results of this analysis; note that the control variables including bicycle speed and latitude/longitude have been omitted from the table for clarity. Overall, the models explained between 67.4 and 70.1 percent of the variation in air temperature. While the model with a 500 m radius of land cover had the highest R^2^ at 0.701, there was only one model in which all of the dependent variables were statistically significant and the coefficient was in the expected direction: the model that used the upwind ellipse in the calculation. This model explained 68.0% of the variation in air temperature. In addition, the F-statistic was higher for the ellipse model than the 500 m radius model.

These findings confirm that the ellipse shape was the best fit for the OLS regression model for three reasons. First, while land covers are somewhat homogenous around each observation point, they are not necessarily symmetrical upwind and downwind of each observation. For example, the Cuyahoga River and Ohio and Erie Canal are adjacent to the path along parts of the route; if one used a large radius circle (~500 m), they would be included in almost every observation, which would reduce the statistical power of this independent variable. Second, while a circle takes into account the surrounding source areas of land covers, it is not directional in nature. Because the ellipse varies with wind direction, the regression model “assigns” this warming to a land cover type. Finally, the towpath runs along the southeast edge of the county. Several of the observations fell outside the land cover database provided by the county; this is why the number of observations in Table 6 varies. If the number of all of the land cover observations were consistent, these results might be different; for example, there might be greater variation in R^2^ and F-statistic among the models.

**Table 6:** Comparison of radii used for air temperature OLS regression analysis.

	500 m Radius	400 m Radius	300 m Radius	200 m Radius	100 m Radius	50 m Radius	25 m Radius	500 m × 300 m Ellipse
n	263	273	286	435	494	499	503	448
F	52.29	53.43	55.20	75.87	86.85	88.08	88.58	80.31
Adjusted R^2^	0.701	0.698	0.695	0.674	0.676	0.677	0.677	0.680
	β	β	β	β	β	β	β	β
Reference Air Temperature	0.454 ***	0.460 ***	0.468 ***	0.476 ***	0.474 ***	0.474 ***	0.473 ***	0.479 ***
Ground Surface Temperature	0.019 ***	0.019 ***	0.020 ***	0.020 ***	0.020 ***	0.020 ***	0.021 ***	0.020 ***
Fraction Forest	−0.512	−0.447	−0.311	−0.367 **	−0.218	−0.166 **	−0.079 ***	−0.249 *
Fraction Water	0.180	−0.521	−0.479 **	−0.264	−0.429	−0.221	−0.032	−0.677 **

* *p* < 0.025, ** *p* < 0.01, *** *p* < 0.001.

### 3.4. Discussion

Incoming solar radiation, albedo, and sky view factor were hypothesized to explain the variation in ground surface temperatures along each transect. An OLS regression model that incorporated spatial effects to control for autocorrelation was found to be statistically significant. The results indicate that for a 600 W/m^2^ reduction in incoming solar radiation, roughly equivalent to being in the shade instead of the full sun, there would be an 8.4 °C drop in the ground surface temperature. Solar radiation ranged from approximately 100 to 900 W/m^2^ on the towpath transects. For every 10% increase in albedo, roughly the difference between asphalt paving and concrete, there was a corresponding 3.4 °C drop in the ground surface temperature. On the towpath rides, variations in solar radiation and albedo led to greater-than −30 °C shifts in the ground surface temperature.

Although sky view factor is frequently mentioned in studies of the UHI effect, it was not found to be statistically related to ground surface temperature in the model. This may be because solar radiation and sky view factor were positively correlated, and therefore one of the two variables had to drop from the model. It may also be due to the unique nature of the towpath route, which passed through low-rise commercial/industrial spaces and natural areas as opposed to a typical downtown.

Second, it was hypothesized that reference air temperature, ground surface temperature, and land cover types would explain the variation in air temperatures along each transect. An OLS regression model that incorporated spatial effects to control for autocorrelation was found to be statistically significant. The results indicate that for a 10 °C increase in the ground surface temperature, there is a 0.2 °C increase in the local air temperature. This demonstrates a link between local ground surface temperatures and air temperature; the selection of paving materials has a significant effect on both surface and atmospheric UHIs. However, one might expect this relationship to be stronger; the ratio relating air and ground surface temperature would likely change if data was taken on days with a higher air temperature (>29 °C). These results may also indicate that waste heat from industrial facilities or highways may play a larger role than expected in determining local air temperatures, consistent with Coseo and Larsen [4].

Temperatures recorded downwind from a forest were 0.25 °C cooler than those recorded over impervious, bare soil, or grass land covers. Water also provided a cooling effect that was roughly 2.7 times stronger than that of a forest. However, very large areas of forest or water were necessary to achieve a drop in the local air temperature; roughly 11.8 hectares of water (29.16 acres) produced only a 0.67 °C drop in the local air temperature. These results help to understand how land cover may impact human exposure to temperature; with these results the city or county can direct tree planting programs in areas with lower tree canopy or high quantities of impervious surfaces. They may also choose to focus on heat mitigation programs in neighborhoods further away from the protective cooling of Lake Erie.

Overall, the bicycle performed well as a platform to gather data to analyze ground and surface temperatures. It allowed one to reach locations that would be inaccessible by automobile and was less expensive than setting up multiple research-grade weather stations. In addition, riding a bicycle helped in the validation of land cover data, something that would be difficult to accomplish from the confines of an automobile. Using a bicycle may also improve the validity of results because the data are not subject to contamination from vehicle exhaust.

However, bicycles do have significant limitations, such as safety concerns, heat stress, rider fatigue, and difficulty scaling steep terrain. Interpretation of weather data from mobile measurement systems is also more difficult than interpreting results from static weather stations, though collecting data with one set of sensors avoids the need to cross calibrate equipment. Although the cost of building the bicycle is lower than purchasing multiple research grade weather stations, or getting custom remote sensing data from satellites or airplanes, the cost is still significant. Future work could other setups to gather mobile data, like Brandsma and Wolters [35] who simply attached a datalogger to a bicycle, or using other low-cost datalogging systems to produce a similar analysis.

#### Limitations of the Study

Like any study, this research has a number of limitations. First, due to a Scan Line Corrector failure on Landsat 7 the satellite imagery originally intended for land cover analysis contained significant gaps. An estimated 22 percent of each scene was lost; the data gaps occur along the edge of each image acquired by the satellite [47]. While it is possible to patch together one or more images to create complete scenes, most of the Landsat 7 images taken during the summer of 2012 also had cloud cover that obscured the land cover below, making the patching process unreliable. For these two reasons, the Cuyahoga County’s 2011 land cover classification was used. While the classification was consistent with current aerial photographs and the experience riding along the canal, in future investigations of microclimates it would be helpful to have up to date land cover data since it may vary year to year.

Second, it would be helpful to ride the bicycle more times and with a higher temporal resolution to establish strong statistical relationships among all of the data. As a comparison, Brandsma and Wolters [35] completed 183 transects along a 14-km route in Utrecht over the course of three years, taking air temperature and relative humidity measurements every second. For future studies, the plan is to consolidate rides on one path with the widest range of land covers, such as the Canal Towpath, and to take measurements on a wide range of high heat days. The temporal resolution of the data will also be increased to once per second, and the bicycle will be ridden at night to determine the maximum heat island effect.

## 4. Conclusions

Assessing human exposure to high temperature will require understanding both how average temperatures are expected to shift with global warming and how local temperatures are modified by the urban heat island effect. Although airport weather stations and remote sensing all help to estimate ground surface and air temperatures in a city, finer scale data is needed to support preventative programs at the neighborhood-level like cooling centers or the planting of street trees to reduce temperatures.

Using a bicycle, a fine scale of microclimate data was collected to determine how physical characteristics (e.g., solar radiation, albedo, sky view factor, vegetation) contributed to local variations in ground and air temperatures. This bicycle expanded on a methodology used by the building science and urban climate communities. Solar radiation and albedo explain the variation in ground surface temperatures along a transect in Cuyahoga County, Ohio. In turn, the ground surface temperature and land cover types explained the variation in air temperatures.

Bicycles are a viable and low-cost alternative to stationary weather stations, microdataloggers, and automobile-based measurements of microclimates. Because of its relatively low cost, city planners may want to develop similar systems to estimate exposure in their own cities. However, additional work is needed to standardize measurement protocols to allow for comparative studies.

## References

[1] Melillo J.M., Richmond T., Yohe G.W. (2014). Climate Change Impacts in the United States: The Third National Climate Assessment.

[2] Imhoff M.L., Zhang P., Wolfe R.E., Bounoua L. (2010). Remote sensing of the urban heat island effect across biomes in the continental USA. Remote Sens. Environ..

[3] Oke T.R., Thompson R.D. (1997). Urban climates and global environmental change. Applied Climatology: Principles & Practices.

[4] Coseo P., Larsen L. (2014). How factors of land use/land cover, building configuration, and adjacent heat sources and sinks explain urban heat islands in chicago. Landsc. Urban Plan..

[5] Harlan S.L., Brazel A.J., Prashad L., Stefanov W.L., Larsen L. (2006). Neighborhood microclimates and vulnerability to heat stress. Soc. Sci. Med..

[6] Sheridan S.C. (2002). The redevelopment of a weather-type classification scheme for north america. Int. J. Climatol..

[7] Oke T.R. (2006). Initial Guidance to Obtain Representative Meteorological Observations at Urban Sites.

[8] White-Newsome J.L., Brines S.J., Brown D.G., Dvonch J.T., Gronlund C.J., Zhang K., Oswald E.M., O’Neill M.S. (2013). Validating satellite-derived land surface temperature with *in situ* measurements: A public health perspective. Environ. Health Perspect..

[9] Coseo P.J. (2013). Evaluating Neighborhood Environments for Urban Heat Island Analysis and Reduction. Ph.D. Thesis.

[10] Altman P., Lashof D., Knowlton K., Chen E., Johnson L., Kalkstein L. (2012). Killer Summer Heat: Projected Death Toll from Rising Temperatures in America Due to Climate Change.

[11] Reid C., O’Neill M.S., Gronlund C.J., Brines S.J., Brown D.G., Diez-Roux A.V., Schwartz J. (2009). Mapping community determinants of heat vulnerability. Environ. Health Perspect..

[12] Staudt A., Inkley D. (2009). More Extreme Heat Waves: Global Warming’s Wake up Call.

[13] Stewart I.D. (2011). A systematic review and scientific critique of methodology in modern urban heat island literature. Int. J. Climatol..

[14] Manley G. (1958). On the frequency of snowfall in metropolitan england. Q. J. R. Meteorol. Soc..

[15] Voogt J.A., Oke T.R. (2003). Thermal remote sensing of urban climates. Remote Sens. Environ..

[16] Santamouris M. (2001). Energy and Climate in the Built Environment.

[17] EPA (2008). Reducing Urban Heat Islands: Compendium of Strategies.

[18] Oke T.R. (1982). The energetic basis of the urban heat island. Q. J. R. Meteorol. Soc..

[19] Lo C.P., Quattrochi D.A. (2003). Land-use and land-cover change, urban heat island phenomenon, and health implications: A remote sensing approach. Photogramm. Eng. Remote Sens..

[20] Kwok A.G. (1998). Thermal comfort in tropical classrooms. ASHRAE Trans..

[21] Benton C.C., Bauman F.S., Fountain M.E. (1990). A field measurement system for the study of thermal comfort. ASHRAE Trans..

[22] American Society of Heating, Refrigerating and Air-Conditioning Engineers, Inc. (2010). Ansi/Ashrae Standard 55–2010: Thermal Environmental Conditions for Human Occupancy.

[23] Potvin A. (1997). The arcade environment. Archit. Res. Q..

[24] Chun C., Tamura A. (2005). Thermal comfort in urban transitional spaces. Build. Environ..

[25] Unger J., Sümeghy Z., Zoboki J. (2001). Temperature cross-section features in an urban area. Atmos. Res..

[26] Oke T.R. (1973). City size and the urban heat island. Atmos. Environ. (1967).

[27] Hedquist B.C., Brazel A.J. (2006). Urban, residential, and rural climate comparisons from mobile transects and fixed stations: Phoenix, arizona. J. Ariz. Nev. Acad. Sci..

[28] Straka J.M., Rasmussen E.N., Fredrickson S.E. (1996). A mobile mesonet for finescale meteorological observations. J. Atmos. Ocean. Technol..

[29] Conrads L.A., van der Hage J.C.H. (1971). A new method of air-temperature measurement in urban climatological studies. Atmos. Environ. (1967).

[30] Stewart I.D. (2011). Redefining the Urban Heat Island. Ph.D. Thesis.

[31] Melhuish E., Pedder M. (1998). Observing an urban heat island by bicycle. Weather.

[32] Heusinkveld B.G., van Hove L., Jacobs C., Steeneveld G., Elbers J., Moors E., Holtslag A. Use of a Mobile Platform for Assessing Urban Heat Stress in Rotterdam. Proceedings of the 7th Conference on Biometeorology Albert-Ludwigs-University of Freiburg.

[33] Dai Q., Schnabel M.A., Heusinkveld B. Influence of height-to-width ratio: Case study on mean radiant temperature for netherlands buildings. Proceedings of the 46th Annual Conference of the Architectural Science Association (ASA 2012).

[34] Brandsma T., Wolters D. (2012). Measurement and statistical modeling of the urban heat island of the city of utrecht (the Netherlands). J. Appl. Meteorol. Climatol..

[35] National Oceanic and Atmospheric Administration Comparative Climatic Data. http://ols.nndc.noaa.gov/plolstore/plsql/olstore.prodspecific?prodnum=C00095-PUB-A0001.

[36] Perera E.M., Sanford T., White-Newsome J.L., Kalkstein L.S., Vanos J.K., Weir K. (2012). Heat in the Heartland: 60 Years of Warming in the Midwest.

[37] Schoof J.T., Pryor S.C. (2013). Historical and projected changes in human heat stress in the midwestern united states. Climate Change in the Midwest: Impacts, Risks, Vulnerability, and Adaptation.

[38] Clark J.A., Follin G.M. (1988). A simple “equal area” calibration for fisheye photography. Agric. For. Meteorol..

[39] Herbert T.J. (1987). Area projections of fisheye photographic lenses. Agric. For. Meteorol..

[40] Frazer G.W., Fournier R.A., Trofymow J.A., Hall R.J. (2001). A comparison of digital and film fisheye photography for analysis of forest canopy structure and gap light transmission. Agric. For. Meteorol..

[41] Grimmond C.S.B., Potter S.K., Zutter H.N., Souch C. (2001). Rapid methods to estimate sky-view factors applied to urban areas. Int. J. Climatol..

[42] University of Gothenburg Department of Earth Sciences The Skyviewfactorcalculator, Version 1.1. http://gvc.gu.se/english/research/climate/urban-climate/software/skyviewfactorcalculator.

[43] Cuyahoga County GIS Department Cuyahoga County Gis Data. http://gis.cuyahogacounty.us/en-US/GIS-Data.aspx.

[44] NOAA National Data Buoy Center. http://www.ndbc.noaa.gov/station_page.php?station=cndo1.

[45] Immergluck D., Smith G. (2006). The external costs of foreclosure: The impact of single-family mortgage foreclosures on property values. Hous. Policy Debate.

[46] Galster G., Temkin K., Walker C., Sawyer N. (2004). Measuring the impacts of community development initiatives: A new application of the adjusted interrupted time-series method. Eval. Rev..

[47] USA Department of the Interior SLC-Off Products: Background. http://landsat.usgs.gov/products_slcoffbackground.php.

